# Adherence to long-term telemonitoring-supported physical activity in patients with chronic heart failure

**DOI:** 10.1038/s41598-024-70371-0

**Published:** 2024-09-26

**Authors:** Pauline Lödding, Sebastian Beyer, Christoph Pökel, Momme Kück, Christian Leps, Lukas Radziwolek, Arno Kerling, Sven Haufe, Antina Schulze, Stefan Kwast, Johannes Voß, Christian Kubaile, Uwe Tegtbur, Martin Busse

**Affiliations:** 1 Department of Rehabilitation and Sports Medicine, Hannover Medical School, Carl-Neuberg-Str. 1, 30625 Hannover, Germany; 2 Institute of Sports Medicine and Prevention, University of Leipzig, Leipzig, Germany

**Keywords:** Chronic heart failure, Physical activity, Sedentary behavior, Telemonitoring, Digital therapeutics, Exercise intervention, Barriers, Predictors, Cardiology, Health care, Risk factors

## Abstract

Chronic heart failure (CHF) is one of the most common diseases with a prevalence of 1–2% in adults, disproportionately affecting the elderly. Despite consistent drug therapy, physical activity (PA) is an integral part of current guidelines. Yet adherence to regular PA and exercise interventions is poor and potential predictors and barriers to PA remain elusive. We examined the effects of a telemonitoring-based exercise intervention in 699 CHF patients in a prospective, randomized-controlled (1:1), multicenter trial. The study was registered in the German Clinical Trials Register under DRKS00019022 on 28.05.2020. For both, the exercise and control group, self-reported PA (MET*h/week) increased and sedentary behavior declined during the 12-month intervention period. In the exercise group, daily step count as analyzed via activity trackers remained stable (pre: 6459 [4016] steps/day, post: 6532 [3858] steps/day; p = 0.621). The average number of completed exercise instruction videos provided via an online application was 1.50 [1.44] videos/week at the beginning and gradually decreased to 1.00 [1.50] videos/week; p < 0.001). Multivariate regression model revealed that exercise-related PA (MET*h/week) and exercise capacity (W_max_) at baseline, CHF severity, atrial fibrillation and age predicted changes in self-reported exercise-related PA (R^2^ = 0.396). Furthermore, the BMI and the average number of completed videos per week at baseline were associated with the change in completed videos over the course of the study (R^2^ = 0.251). Our results show the influence of certain baseline characteristics as barriers and predictors of PA progression. Therefore, exercise programs should pay attention to patients’ individual conditions to set achievable goals, and eventually affect the adherence and sustainability of exercise-focused interventions.

## Introduction

Chronic heart failure (CHF), in which the heart is no longer able to supply the organism with sufficient blood and oxygen, is one of the most common diseases with a prevalence of 1–2% in developed countries, disproportionately affecting the elderly^1–3^. As studies typically include diagnosed cases of CHF only, the true prevalence is likely to be higher^3,4^. Physical inactivity and sedentary behavior are considered as the leading modifiable risk factors for cardiovascular diseases and all-cause mortality^5^. Overall levels of physical inactivity have reached pandemic proportions worldwide, with more than 25% of all adults failing to meet the World Health Organization (WHO) recommendations for physical activity (PA)^6,7^. Furthermore, adherence to exercise interventions in patients with CHF remains poor; it appears to be even more difficult to achieve than dietary changes and consistent drug therapy^8^. Factors related to this low adherence include chronic comorbidities, little disease-related knowledge and clinical depression but also limited access to cardiac rehabilitation services and lack of social support for CHF patients^9–11^.

In light of this, there is a need for safe and effective strategies, independent of the place of residence, in order to facilitate PA in patients with CHF. A recent review on the safety and long-term outcomes of remote cardiac rehabilitation in patients with coronary heart disease showed that telemedical implementation of cardiac rehabilitation is safe and does not increase the rehospitalization rates^12^. Another review indicated that exercise capacity and health-related quality of life (HrQoL) increased significantly in most studies^13^. Yet, it is important to understand which factors influence or predict long-term PA behavior. For example, chronic heart conditions may serve as both a barrier and a motivation for PA in the older adult population^14^. However, a new diagnosis and being physically active at baseline, and higher levels of education, exercise self-efficacy, and motivation may be significant predictors of frequent program attendance in CHF patients^15,16^. In today’s age of increasing digitalization, telemedical therapy concepts are playing an increasingly important role, benefiting patients and already being used in everyday life. In this context, the aim of the current analysis is, firstly, to evaluate PA in the setting of a telemonitoring-based exercise study in patients with CHF and, secondly, to examine potential predictors and barriers to PA in the long-term. One potential impact on care planning and practice may be that defined predictors and barriers at the onset of a PA intervention may guide the content of the intervention and determine the level of care required to promote sustainable PA in the long-term.

## Materials and methods

### Study design

All participants took part in a prospective, randomized, parallel group, multicenter controlled trial examining the effects of telemonitoring-supported exercise training in patients with CHF. The “HITS” study (“Heart failure, Individual exercise training, Telemonitoring, Selfmanagement”) was conducted as a collaborative project between Leipzig University (LU), Hannover Medical School (MHH), Leipzig Heart Center (LHC), AOKplus health insurances, IGES institute, DiaVention GmbH, and the Clinical Centers in Wolfsburg (CCW) and Chemnitz (CCC) in Germany. The study was registered in the German Clinical Trials Register under DRKS00019022 (28.05.2020). The institutional review boards of the MHH (No. 8786) and the medical faculty of the LU (479/19-ek) have given ethical approval for the study, and written informed consent was obtained prior to enrollment. The HITS innovation fund project aimed to establish a new model of care that included early diagnosis of early stages of CHF, the treatment of CHF patients according to current ESC and AHA guidelines^17,18^, avoidance of hospitalizations, and improvement of treatment adherence, physical performance, and HrQoL.

### Subjects

Participants were recruited through a series of information events, referrals from general practitioners, and newspaper and online advertisements. According to predefined inclusion criteria, patients older than 18 years of age with diagnosed CHF of the stages NYHA I, II, and III (New York Heart Association [NYHA] clinical symptom stages^19^, including transplants or implants were included in the study. Patients with both existing and newly diagnosed CHF were included. For the latter, N-terminal natriuretic pro-B peptide (NT-proBNP) level greater than 125 pg/ml was used as the cutoff for inclusion according to the ESC criteria^18^. Exclusion criteria were the presence of chronic kidney disease, chronic obstructive pulmonary disease, alcohol abuse or use of illegal drugs, active participation in other studies, and any physical or mental condition that precluded participation in an exercise intervention. Participants who did not show up for the examinations or were unwilling to use the study devices were excluded from the study.

**Figure. 1:**
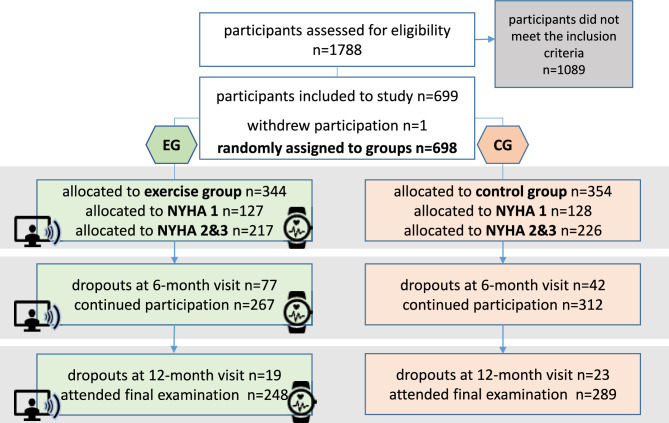
Study design and randomization.

All participants were randomly assigned (at a 1:1 ratio) to the exercise group (EG) or the control group (CG) (see Fig. 1). Classification into NYHA stages I, II, or III was based on existing diagnoses or was determined by the study physicians based on the severity of symptoms during PA^19^.

For the present analysis, available data from all included patients from all study centers were evaluated.

### Procedures

At each study center, anthropometric data (body weight, height, body mass index [BMI], waist and hip circumference) were assessed according to defined standard operation procedures (SOPs) after a general medical examination by a physician (including electrocardiogram, medical history, and physical examination). Fat-free mass, fat mass, and total body water as markers of body composition were estimated by segmental, multifrequency, bioimpedance analysis (MHH: InBody720; Biospace, South Korea; LU. CCC, CCW: Biacorpus RX 4004M; MediCal HealthCare GmbH, Germany).

Cardiac-specific laboratory parameters (NT-proBNP, creatine kinase [CK], isoenzyme creatine kinase-MB [CK-MB], C-reactive protein [CRP], interleukine-6 [IL-6], and a safety blood profile including electrolytes, hemoglobin, hematocrit, thrombocytes, and leucocytes) were obtained from a venous blood sample.

Exercise capacity (measured as peak power output in watts [W_max_]) and maximum heart rate (HR_max_) were assessed with stepwise cardiopulmonary exercise testing (CPET) on a bicycle ergometer (Ergoline P150, Bitz, Germany) until objective or subjective maximal exertion, or pathological findings for ergometry occurred. To ensure the accurate assessment of maximal exertion, we measured blood lactate levels and considered levels > 4 mmol/l as exhaustion. During each CPET, we measured the respiratory exchange ratio (RER) and used an RER value > 1.10 to indicate maximal effort. In each CPET we achieved O_2_-ventilatory equivalent, which can be used as a valid sign of exhaustion when V̇E/V̇O_2_ > 30–35. Unlike healthy individuals and athletes, where VO_2max_ is determined, cardiac patients often do not reach a VO_2_ plateau at the end of a CPET. Therefore, we used the VO_2peak_ value and the maximal wattage as measures of performance. The FRIEND equation^20^ and the Wasserman-Hansen algorithm^21^ were used to determine peak predicted relative VO_2_, and percent peak predicted VO_2_ (ppVO_2_) was calculated as 100 × (measured relative peak VO_2_/peak predicted relative VO_2_). Subjective perceived exertion was assessed by the Borg-Scale^22^. The CPET started with a workload of 20 W or 50 W and was increased in 10 W or 17 W increments per minute. A spirometric system (Oxycon CPX, CareFusion, Würzburg, Germany) was used to measure breath-by-breath oxygen uptake (VO_2_ [ml/min]), carbon dioxide production (VCO_2_ [ml/min]), and ventilation. Heart rate and blood pressure were recorded and capillary blood samples were taken from the earlobe at rest, 1 min after the start, and every 3 min during the test to determine blood lactate concentrations (Ebio 6666, Eppendorf, Germany). Because we did not have the appropriate equipment at the Clinical Center Wolfsburg, we only performed a stepwise exercise test on a bicycle ergometer at this location without additional spirometry.

A comprehensive transthoracic echocardiography was performed in accordance with AHA/ESC guidelines (CCW: Vivid™ T9, GE Healthcare GmbH, Solingen, Germany; MHH: Vivid iq, GE Healthcare GmbH, Solingen, Germany). If an echocardiography was performed less than 8 weeks previously by a resident cardiologist, the echocardiographic values from the physician's report were used.

The description of questionnaires used has been described in detail previously^23^. Briefly, a 14-item questionnaire was distributed as a primary measure to estimate adherence to the Mediterranean Diet (MDS questionnaire)^24^. The 23-item Kansas City Cardiomyopathy Questionnaire (KCCQ) was used to quantify physical limitations, symptoms, self-efficacy, social interference and HrQoL in patients with CHF^25^. The Sedentary Behavior Questionnaire (SBQ) was used to assess the amount of time spent in nine behaviors (watching television, playing computer/video games, sitting while listening to music, sitting and talking on the phone, doing paperwork or office work, sitting and reading, playing a musical instrument, doing arts and crafts, sitting and driving/riding in a car, bus, or train). The nine items were completed separately for weekdays and weekend days^26^. The results refer to the total number of hours spent in sedentary behavior during a 7-day week. At the study centers MHH and CCW, the Freiburger PA Questionnaire was distributed to calculate the total and exercise-related PA as metabolic equivalents of task (MET) hours per week^27^. All questionnaires and examinations were carried out at the 6-month (V6) and the 12-month (V12) visits according to the baseline visit (V0).

### Intervention

After randomization at V0, participants in the EG were provided with the following study devices:

- wearable activity tracker with accelerometer and GPS sensors (vivoactive 4, Garmin®, Garching, Germany) to record heart rate and daily step count,
- heart rate monitor (HRM-DUAL, Garmin®, Garching, Germany) to record heart rate during exercises,
- upper arm blood pressure monitor (boso medicus system, Bosch & Sohn GmbH, Jungingen, Germany) to measure resting blood pressure,
- personal scale (Trisa Electronics, Body Analyze 4.0, Triengen, Switzerland) to measure body weight,
- tablet computer (Lenovo® TB-X606X, Lenovo® Germany, Stuttgart, Germany) as an interface between the devices.

After testing the devices with the assistance of the study's sports scientists, EG participants were introduced to the study application, called the “HITS App”, which was installed on the tablet. The HITS App is a native mobile application for android operating system. The user interface has been optimized for tablets. The App was developed by the DiaVention GmbH for the HITS study and included the following components: Home-based exercise training, information on general health improvement, disease prevention, and lifestyle changes (diet, exercise, and self-awareness). All measurement data transmitted by the devices via Bluetooth was summarized and graphically processed in the App. Both participants and study staff had access to the daily measured data. This feature helped to actively engage patients in the intervention and allowed the sports scientists to adjust the intervention at any time if necessary. Most importantly, the App served as a platform for the home-based exercise training videos, which were selected based on group affiliation (NYHA I, II, or III) and/or individual limitations. The videos averaged 30 min in length and were interval training in nature. The intensity of the exercises bodyweight training should be in the moderate to vigorous range. Based on the results of the CPET, a symptom-limited upper limit of heart rate was set by the physicians. The content included exercises to improve aerobic endurance as well as strengthening exercises for the trunk, upper and lower extremities. Throughout the intervention, the task was to maintain PA at the highest level possible, aiming for three exercise videos per week, taken into account individual limitations and preferences. Participants in the EG were instructed to measure their resting blood pressure and body weight on a daily basis as well as to answer questions about adherence to drug therapy and their sleep quality, and to transmit data from the wearable activity tracker to the App. All EG participants were given a wearable activity tracker at baseline to determine PA via steps per day and moderate-to-vigorous physical activity (MVPA) per week. All indoor and outdoor activities, that were done additionally to the exercise training videos via the App, could be recorded on the activity tracker and were presented in steps per day and MVPA.

To evaluate participants’ adherence to the exercise recommendations, the average number of completed exercise videos per week and the daily step count via the wearable activity tracker at V0 and at V12 was analyzed. For both parameters, a minimum of 13 valid and continuous weeks had to be recorded. We considered a daily step count of at least 500 steps as a valid day. We chose the delta of the last 4 weeks before the final exam and the first 4 weeks after the devices were issued as the dependent variable in a multivariate linear regression model. In order to be able to evaluate the progress and the adherence to the 12-month exercise intervention, we compared the average number of completed exercise videos in the first 4 weeks with the average number in the last 4 weeks. Data of the wearable activity tracker were used to determine the daily step count and the heart rate throughout the day. Since these data are only available for the EG, the Freiburger PA questionnaire was used to assess and compare self-reported PA over time and between EG and CG. To assess the sedentary behavior among study participants, the SBQ was evaluated at baseline and throughout the study.

### Statistical analysis

First, the Kolmogorov–Smirnov test was used to test for normal distribution. Differences between the two groups were compared using the Mann–Whitney-U-Test (not normally distributed data), the Student t-test for unpaired samples (normally distributed data), or the chi-square test for frequency distributions, using Pearson's correlation coefficient r as the effect size for the Mann–Whitney U test, Cohen’s d for the Student t-test, and Cohen’s ω for the chi-square test. An effect size of 0.1 indicates a small effect, 0.3 indicates a medium effect, and 0.5 indicates a large effect. Parametric values were reported as mean and standard deviation (SD); non-parametric values were reported as median values and interquartile range [IQR]. For descriptive analysis, absolute frequencies were calculated for categorical variables, and mean and SD for continuous variables. Univariate correlations between parameters were tested using Pearson’s or Spearman’s correlation coefficient depending on normality of data distribution.

Regression analyses were performed to identify parameters associated with changes in PA (MET-hours per week by questionnaire), everyday activity (step count per day by wearable activity tracker) and number of training videos (recorded by HITS App) after the 12-month intervention.

A one-way analysis of variance (ANOVA) was used to test for group differences between the subscales of the Freiburger PA Questionnaire and the SBQ at baseline, using eta squared η^2^ as the effect size, where 0.01 indicates a small effect, 0.06 indicates a medium effect, and 0.14 indicates a large effect. Post hoc tests were corrected according to Bonferoni. An effect size is only reported if the corresponding test was significant. All statistical analyses were carried out *per protocol*. The type-I-error was set to 5% (two-sided). All statistical analyses were performed using IBM SPSS 28 Statistics (IBM Corporation, NY, USA).

### Ethics approval and consent to participate

The study was performed in accordance with the Declaration of Helsinki and current guidelines of good clinical practice. The study was completed as a cooperation project between Leipzig University, Hannover Medical School, Leipzig Heart Center, AOKplus health insurances, IGES institute, DiaVentions GmbH, and the Clinical Centers in Wolfsburg, Chemnitz and Dresden (Germany). The study is registered under DRKS00019022 in the German Clinical Trials Register. The institutional review boards of Hannover Medical School (No. 8786) and medical faculty of the Leipzig University (479/19-ek) ethical approved the study, and written informed consent was obtained prior to inclusion of study participants.

## Results

### Participants’ characteristic

The combined dataset consists of 698 participants, of whom 537 completed the 12-month study. We registered 162 drop-outs during the course of the study. Table 1 shows subject characteristics at V0 for anthropometric and exercise related parameters, laboratory markers, distribution of comorbidities, and capacity of the heart as well as exercise-related, dietary-related, and health-related outcomes by questionnaire. The RER was not significantly different between EG and CG (EG: 1.20 [0.11], CG: 1.23 [0.17], p = 0.137).

**Table 1:** Subject characteristics at baseline.

	EG	n	CG	n	*p*-value	Effect size
Antropometric data						
Sex (men [%]/women [%])	195 [56.7%]/149 [43.3%]		221 [62.4%]/133 [37.6%]		0.122	–
Age [years]	68 [14]	344	69 [15]	354	0.285	–
NYHA I [%]	127 [36.9%]		128 [36.2%]		0.835	–
NYHA II & III [%]	217 [63.1%]		226 [63.8%]			
Body weight [kg]	81.9 [21.9]	344	82.0 [20.9]	354	0.468	–
Body mass index (BMI [kg/m^2^])	27.3 [6.4]	344	27.5 [6.1]	354	0.950	–
Body fat [kg]	23.9 [13.7]	271	25.0 [15.1]	274	0.664	–
Fat free mass [kg]	56.0 [19.7]	271	57.5 [18.1]	274	0.475	–
Waist circumference [cm]	100.2 ± 14.5	318	100.1 ± 14.2	323	0.893	–
Hip circumference [cm]	104.0 [13.0]	312	105.0 [12.0]	317	0.962	–
Waist-to-hip-ratio	0.96 [0.12]	312	0.96 [0.12]	317	0.847	–
Pacer [%]	10.8	344	8.5	354	0.306	–
Ejection fraction						
LVEF BiPQ [%]	60.0 [13.8]	176	60.0 [16.0]	190	0.661	–
LVEF (% with normal LVEF)	65.4	240	70.7	242	0.608	–
LVEF (% with slightly restricted LVEF)	23.8	240	19.0	242		
LVEF (% with moderate restricted LVEF)	7.5	240	7.4	242		
LVEF (% with highly restricted LVEF)	3.3	240	2.9	242		
Exercise capacity						
Exercise capacity (W_max_ [W])	110.0 [50.0]	335	110.0 [50.0]	348	0.514	–
Relative exercise capacity [W/kg]	1.35 [0.59]	335	1.37 [0.61]	348	0.952	–
Maximum oxygen uptake (VO_2peak_ [ml/min])	1702.5 [794.3]	266	1620.5 [673.2]	274	0.849	–
Relative oxygen uptake (VO_2peak_ [ml/min/kg])	20.75 [8.38]	266	20.1 [7.9]	274	0.514	–
Percent peak predicted VO_2_ (ppVO_2FRIEND_ [%])	89.2 [37.6]	266	86.3 [36.1]	274	0.650	–
Percent peak predicted VO_2_ (ppVO2_WH_ [%])	91.8 [41.1]	266	92.4 [36.6]	274	0.692	–
Laboratory markers						
NT-proBNP [ng/l]	244.0 [303.0]	341	249.0 [299.8]	352	0.671	–
IL-6 [ng/l]	3.0 [1.6]	318	3.3 [1.5]	319	0.240	–
CRP [mg/l]	1.4 [2.3]	334	1.4 [2.4]	346	0.933	–
Comorbidities						
Coronary Artery Disease [%]	33.4	344	36.4	354	0.404	–
Atrial fibrillation [%]	23.3	344	28.0	354	0.154	–
Hypertension [%]	64.5	344	70.6	354	0.086	–
Type 2 Diabetes [%]	11.0	344	16.4	354	**0.041**	0.08
Questionnaires						
Total PA [MET*h/week]	32.2 [42.9]	127	28.9 [33.8]	136	0.542	–
MDS total score	6.0 [3.0]	282	6.0 [4.0]	197	**0.035**	0.10
KCCQ overall Score	80.2 [25.2]	301	80.2 [23.0]	321	0.926	–
KCCQ symptom Score	85.7 [25.0]	297	85.7 [28.3]	319	0.389	–
KCCQ functional Score	84.7 [23.8]	297	84.7 [23.8]	319	0.527	–
KCCQ clinical summary Score	82.3 [25.9]	301	82.8 [24.0]	321	0.772	–

Significant values are in [bold].

Parametric values were reported as mean and SD, non-parametric values were reported as median and interquartile range.

PA, physical activity; KCCQ, Kansas City Cardiomyopathy Questionnaire; MDS, Mediterranean Diet Score; LVEF, left ventricular ejection fraction; BiPQ, biplane method (Simpson); ppVO_2FRIEND_, percent peak predicted VO_2_ by the FRIEND equation; ppVO_2WH_, percent peak predicted VO_2_ by the Wasserman-Hansen algorithm.

### Sedentary behavior by questionnaire

Table 2 shows the results of the SBQ in total hours spent in sedentary activity during a 7-day week. For both EG and CG, sedentary behavior decreased from V0 to end of study V12, but with no group difference.

**Table 2:** Changes in sedentary behavior by questionnaire over time and between the study groups in total hours in a 7-day week.

Group	n	SBQ visit 0	SBQ visit 6	SBQ visit 12	Time		Time × group	
					p_Anova_	η^2^	p_Anova_	η^2^
EG	86	56.2 ± 19.0	54.4 ± 18.8	52.9 ± 17.3	**0.027**	0.02	0.303	–
CG	103	55.9 ± 19.9	57.8 ± 21.9^a^	53.6 ± 20.8^a^				
total	189	56.1 ± 19.4^b^	56.3 ± 20.5	53.3 ± 19.2^b^				

Significant values are in [bold].

SBQ, sedentary behaviour questionnaire. Total hours spent in sedentary work in a 7-day week.

η^2^ = effect size eta squared.

^a^p < 0.05 visit 6 vs visit 12.

^b^p < 0.05 visit 0 vs visit 12.

### Physical activity by questionnaire

For 188 out of 355 subjects (centers MHH + WOB), data for the Freiburger PA Questionnaire were available, for which a delta of self-reported PA (end minus start of intervention) can be calculated. The EG and CG in this subgroup did not differ in age (EG: 64 years, CG: 66 years, p = 0.053), exercise capacity (W_max_: EG: 121.7 W, CG: 125.9 W, p = 0.443), and in terms of the severity of the disease (NYHA I EG: 30.7%, NYHA II & III EG: 69.3%; NYHA I CG: 34.7%, NYHA II & III CG: 65.3%, p = 0.562). Table 3 shows the results of the Freiburger PA Questionnaire for total, exercise-related, every day, and basic PA in MET-hours per week for this subgroup.

**Table 3:** Changes in basic, everyday, exercise-related, and total PA in MET-hours per week over time between the study groups.

	Group	n	Visit 0	Visit 6	Visit 12	Time		Time × group	
						p_Anova_	η^2^	p_Anova_	η^2^
Basic PA [MET*h/week]	EG	88	14.0 ± 13.5	11.1 ± 12.6^b^	16.2 ± 14.0^b^	** < 0.001**	0.10	0.590	–
	CG	101	13.6 ± 12.2^a^	12.7 ± 13.9^b^	18.0 ± 17.5^a,b^				
	Total	189	13.8 ± 23.8^a,c^	12.0 ± 13.3^b,c^	17.2 ± 16.0^a,b^				
Everyday PA [MET*h/week]	EG	88	15.5 ± 19.9	16.7 ± 20.4	17.9 ± 18.3	0.483	–	0.766	–
	CG	101	16.7 ± 20.1	15.7 ± 14.8	17.5 ± 22.0				
	Total	189	16.1 ± 20.0	16.2 ± 17.6	17.7 ± 20.3				
Exercise-related PA [MET*h/week]	EG	88	7.0 ± 12.9	6.0 ± 7.6	8.3 ± 11.8	**0.043**	0.02	0.924	–
	CG	101	8.6 ± 17.6	7.4 ± 9.1	10.4 ± 15.1				
	Total	189	7.9 ± 15.5^a^	6.8 ± 8.5	9.4 ± 13.7^a^				
Total PA [MET*h/week]	EG	88	36.5 ± 31.3^a^	33.9 ± 30.7	42.4 ± 29.6^a^	** < 0.001**	0.04	0.955	–
	CG	101	39.0 ± 30.9	35.8 ± 26.4^b^	45.9 ± 41.5^b^				
	Total	189	37.8 ± 31.1^a^	34.9 ± 28.4	44.3 ± 36.4^a^				

Significant values are in [bold].

PA, physical activity; η^2^, effect size eta squared.

^a^p < 0.05 visit 0 vs visit 12.

^b^p < 0.05 visit 6 vs visit 12.

^c^p < 0.05 visit 0 vs visit 6.

Basic PA, exercise-related PA, and total PA (MET*h/week) increased from V0 to V12, with no group differences between CG and EG (Table 3). Everyday PA remained stable throughout the intervention, with no group difference as well.

### Regression models. Changes in exercise-related PA by questionnaire

In a multivariate linear regression model we analyzed the influence of age, sex, BMI, CHF severity, baseline values of NT-proBNP, KCCQ overall score, exercise capacity (W_max_), exercise-related PA (MET*h/week) by questionnaire (Freiburger PA Questionnaire), and the presence of atrial fibrillation and coronary artery disease on the change of exercise-related PA by questionnaire (V12 minus V0). It turned out that exercise related activity (MET*h/week) at baseline (r = − 0.583; β = -– 0.620), exercise capacity (W_max_) at baseline (r = 0.058, β = 0.150), CHF severity (r = 4.599; β = 0.145), atrial fibrillation (r = − 4.770; β = − 0.136), and age (r = 0.181, β = 0.127) have a significant influence on the changes in self-reported exercise-related PA by questionnaire. This regression model can explain 39.6% of the variance in the change in questionnaire-estimated exercise-related PA.

To identify possible factors influencing changes in total PA by questionnaire, we chose the same regression model with the respective baseline value as independent variables. The results of the regression analysis show that only the baseline values of total PA (MET*h/week) (r = − 0.408; β = − 0.373) have a significant influence on the change in total PA by questionnaire. This regression model explains 13.5% of the variance in the change of total PA by questionnaire.

### Exercise videos

For 220 out of 344 participants of the EG, data was available for the analyses of the number of training videos completed at the beginning of the intervention (first 4 weeks) and the average number of training videos completed at the end of the intervention (last 4 weeks). The number of completed videos decreased towards the end of the intervention (pre: 1.50 [1.44] videos/week, post: 1.00 [1.50] videos/week; p < 0.001, r = 0.37).

In a multivariate linear regression model, we analyzed the impact of sex, age, BMI, CHF severity, baseline values of NT-proBNP, KCCQ overall score, exercise capacity (W_max_), number of comorbidities, the left ventricular ejection fraction by category (normal, slightly -, moderately-, severely restricted), and the number of completed videos at the start of intervention (first four weeks) on the change in completed exercise videos (V12 minus V0). The results of the regression analysis show that the number of completed videos per week at the start of the intervention (r = − 0.508; β = − 0.515) and BMI at baseline (r = − 0.031; β = − 0.144) have a significant influence on the change in completed videos. This regression model can explain 25.1% of the variance in the change of completed exercise videos.

### Daily step count

For 279 out of 344 participants of the EG, data was available for the analyses of the step count per day at the beginning of the intervention (first four weeks) and the step count per day at the end of the intervention (last four weeks). The daily step count remained stable towards the end of the intervention (pre: 6459 [4016] steps/day, post: 6532 [3858] steps/day; p = 0.621).

A regression model with the same independent variables at V0 and the respective baseline value on the change in daily step count as the dependent variable yielded only such small results that further consideration would not be purposeful (R^2^ = 0.110).

## Discussion

To evaluate the adherence of an online-supported exercise program and to estimate PA behavior in patients with CHF, we analyzed the questionnaire-estimated PA, activity data assessed by wearable activity tracker, and exercise videos by online application. Our analyses show that both physiological and performance-related parameters (exercise capacity, CHF severity, atrial fibrillation) and anthropometric factors (BMI, age) have an impact on long-term PA behavior in patients with CHF.

The results of our multivariate linear regression model show that among others, older age has a positive effect on questionnaire-estimated exercise-related PA (measured by Freiburger PA Questionnaire). This goes in line with previous studies that have identified age as an important factor in the increasing importance of physical and mental health and performance over the lifespan^28–30^. We assume that there is a high intrinsic motivation among our participants to positively influence their physical health through PA. Especially, since our results revealed that a higher NYHA classification as an expression of a more severe CHF also impacts the changes in exercise-related PA positively. Higher age and the severity of the disease seem to emphasize the urgency of changing exercise behavior for patients with CHF for the better. On the contrary it is known that an overall better health status in CHF patients is associated with higher adherence to an exercise program^31^. Yet, baseline values of the KCCQ in our patients did not influence changes in questionnaire-estimated exercise-related PA, daily step count, or completed exercise videos.

Although women with CHF are supposed to have a higher motivation for PA than men (16), sex had no effect on exercise and activity behaviors in our study. These findings suggest that the care concept of the study could be applied regardless of sex-related differences or self-reported health status at the start of an intervention.

We observed that the presence of atrial fibrillation has a negative influence on changes in exercise-related PA and can therefore be considered as a barrier to long-term PA. Patients with this pathology may require closer supervision in order to maintain PA on a high level. Yet, the influence of atrial fibrillation on PA is not conclusively clarified and the potential relevance for patients with CHF with preserved ejection fraction needs to be further investigated^32^.

In our patients, a higher BMI at baseline had a negative impact on the change in completed exercise videos during the intervention. Accordingly, patients with CHF and an elevated BMI should be monitored more closely and tailored to their individual needs in order to establish a high activity level and to promote weight loss. Indeed, engaging in PA seems to be more challenging for individuals with a higher BMI^33^.

The study was conducted during September 2020 (first patient in) and December 2022 (last patient out), which corresponds to the period of the coronavirus disease 2019 (COVID-19) pandemic. During the COVID-19 pandemic, it has become difficult to provide center-based cardiac rehabilitation for CHF patients. A systematic review on the impact of COVID-19 on social isolation, lifestyle changes, and restrictions showed a significant reduction in PA levels, leading to a decline in physical fitness and an increase in sedentary lifestyle in elderly people^34^. Our results of the PA questionnaire showed a significant increase in total PA, exercise-related PA and basic PA. Jafri et al. showed an improvement of functional outcomes in heart failure patients in a home-based setting in HFrEF and HFpEF patients^35^. In contrast to our study, they included a smaller sample size of 188 patients and carried out a shorter intervention period of 9 months. This study was done in a retrospective setting analyzing who completed the home-based intervention; therefore compliance to PA was not confounding the results. Prospective studies in a telehealth intervention setting including patients at risk for heart failure, like type 2 diabetes^36^ and COPD^37^ also showed an improvement in functional capacity. Our study results go in line with these findings therefore we consider our intervention an efficient strategy to successfully promote PA regardless of the patient’s residence or external circumstances. In larger study cohorts, the management of participants is much more complicated, as usability and barriers with technical devices affect the study outcome^38^. Prediction of long-term PA, as provided by our study, identifies confounders and critical patients group to optimize intervention in the future.

For the subscales exercise-related PA and basic PA of the PA Questionnaire, we could observe a significant increase in PA only between baseline and month 12. The results for the total group in the SBQ questionnaire also showed that sedentary behavior decreased between V0 and V12, but not between V0 and V6. These results suggest that the duration of the 12-month exercise intervention appears to be necessary to establish PA in the long-term. In a previous telemonitoring-supported exercise intervention study with participants with Metabolic Syndrome we could achieve beneficial health outcomes and an increased activity behavior after 6 months already^39^. We encourage further researches to investigate whether the later effect in terms of increased activity behavior in patients with CHF is due to the age of the participants, their affinity for digital applications, or the lack of personal assistance during the intervention period.

In all of our regression models with the dependent variables total PA, exercise-related PA, changes in exercise videos, and changes in daily step count, the respective baseline values have the greatest influence on the changes in exercise behavior. In all cases, a higher baseline value negatively affected the progression of PA during the course of the study. The initially high activity level of our partly very motivated patients could not be maintained in the long run. Rather than interpreting these findings as barriers to PA, we suggest that an already high score in PA at baseline leaves only littles scope for increase. Nevertheless, taking baseline values into account can help identify individuals who are likely to respond positively to the intervention and those who may need additional support or alternative approaches.

Our study has strengths and limitations. We had to record some cancellations or postponements of examinations because patients refused to come to a hospital for the examinations due to the pandemic and the risk of infection, or the facilities were closed for outpatient examinations. As a result, the 6-month interval between examinations could not be observed in all cases. The use of the study devices had to be practiced with participants of the EG, in some cases with high time expense, since the participants often had little or no experience with digital media. This may have resulted in incomplete data for some variables recorded with the activity tracker or the HITS App. Further, the activity tracker did not record performed sport units, including bicycle rides or rehabilitation sports. Hence, these activities were often carried out by the participants, but could only be included in the evaluation as steps per day, MVPA, or by PA Questionnaire at MHH and Clinical Center Wolfsburg (CCW). Since there were already a large number of questionnaires in the study, we had to carefully consider which ones we wanted to hand out in addition to the study protocol in order to keep the response rate high. MHH and CCW chose to add the PA questionnaire, so data were only available from these two study centers (n = 355) instead of the entire study population (n = 699). In general, severe CHF symptoms, other chronic diseases or physical disabilities may limit a patient's ability to participate in and adhere to an exercise program. Anxiety, depression and lack of motivation are common in patients with CHF and can hinder participation in rehabilitation programs. Limited access to healthcare facilities, financial constraints, lack of social support and low levels of education can all act as barriers to regular participation in exercise and activity interventions. We assume that unfamiliarity with digital tools and technologies can be a significant barrier, especially for older adults who may not be comfortable at using modern devices. Inadequate integration of telemonitoring and digital health tools into standard care practices, lack of personalized tracking, and inadequate training of healthcare providers on these technologies may limit the effectiveness of such interventions. Nevertheless, we would like to encourage healthcare providers to offer broad services that support patients with CHF via digital exercise concepts.

The inclusion of 699 patients from multiple centers improves the generalizability, statistical power, and reliability of the study results. As a prospective, randomized controlled trial, the design helps to minimize bias and allows for a clear comparison between the exercise intervention group and the control group. The integration of telemonitoring and digital tools (such as activity trackers and an online application) to monitor exercise adherence and physical activity is a modern approach that allows for continuous, real-time data collection and personalized adjustment of the intervention. The successful use of digital tools and telemonitoring demonstrates that the integration of technology into cardiac rehabilitation programs can be effective, particularly in promoting patient empowerment and providing continuous monitoring and support.

## Conclusions

The study emphasizes the importance of personalized exercise programs. By understanding individual baseline characteristics such as exercise capacity, heart failure severity, BMI, and the presence of atrial fibrillation, healthcare providers can tailor interventions to maximize adherence and effectiveness. The findings suggest that structured, telemonitoring-supported PA interventions can help increase activity levels and reduce sedentary behavior in patients with CHF, which is critical for improving overall health. The results of our evaluations help to understand that barriers and predictors for PA progression during the course of a telemonitoring-supported exercise intervention study in patients with CHF are not always related to their disease or comorbidities. It is however important to tailor rehabilitation and exercise programs to patients’ individual needs and baseline status. Understanding around the influence of certain baseline values helps to predict outcomes and set achievable goals, ultimately impacting the success and sustainability of the intervention.

## Data Availability

The datasets used and/or analyzed during the current study are available from the corresponding author on reasonable request.
